# A cross-sectional survey to evaluate the status of digestion in rotating- and fixed-shift workers

**DOI:** 10.3389/fmed.2026.1850861

**Published:** 2026-07-06

**Authors:** Lekshmy Madhavan Potti Lathadevi, Haritha Chandran, Haroon Irshad, Leena Parameswaran Nair

**Affiliations:** 1 Department of Maulika Siddhanta (Basic Principles of Ayurveda), Amrita School of Ayurveda, Amrita Viswavidyapeetham, Amritapuri, India; 2 Department of Ayurveda Samhita Siddhanta, Sri Dharmasthala Manjunatheswara college of Ayurveda and Hospital, Hassan, Karnataka, India

**Keywords:** circadian rhythm, digestive fire, Jaṭharāgni, shift work, status of Jaṭharāgni

## Abstract

**Background:**

In the sustainment of health, the role of a well-functioning gut and effective digestion in sustaining health and contributing to disease development is immensely important. According to Ayurveda, digestive function is governed by Jaṭharāgni (digestive fire), and an impaired Jaṭharāgni (Mandagni) is considered a key factor in the initiation of many diseases. There are four types of expression of digestive fire (Jaṭharāgni) based on the predominance of the three humors, such as Vishamāgni (irregular digestion), 2. Tīkṣṇāgni (excessively intense digestion), 3. Mandāgni (slow digestion), and 4. Samāgni (balanced digestion). The state of Jaṭharāgni is influenced by several physiological factors, including season, time of day, age, constitution, dosha balance, and the quality and quantity of food consumed. Among shift workers, especially those engaged in rotating shifts, maintaining a regular lifestyle and dietary habits is often challenging. Disrupted sleep–wake cycle and an altered diet habit adversely affect normal gut physiology and impair digestion, metabolism, and gut transit time. The rising importance of shift work in modern society and an increase in the prevalence of metabolic and gastrointestinal disorders associated with the disruption of circadian rhythm highlight the need for research on digestive health among shift workers. Understanding the scientific framework on the relationship between Jaṭharāgni and altered work shifts and suggesting balanced lifestyle practices may contribute to the prevention of bad impacts on metabolic and digestive health.

**Materials and methods:**

A comparative, cross-sectional study was conducted among Information Technology (IT) employees from various companies in Technopark, Thiruvananthapuram, to assess the status of Jaṭharāgni among fixed-shift and rotating-shift workers. The sample size was calculated using Nassiuma’s formula, and 350 employees were selected, comprising 175 fixed-shift workers and 175 rotating-shift workers (alternating between day and night shifts monthly) using a convenience sampling technique. Agnibala was assessed using a validated questionnaire published by Aparna Singh, Girish Singh, Kishore Patwardhan, and Sangeeta Gehlot, Department of Kriya Śarīra, Banaras Hindu University (BHU), Varanasi, India (2016 May) (27381899; PMCID: PMC 5871217). The questionnaire was validated using a Cronbach’s alpha of 0.73 for the overall scale. For fixed-shift workers, Agnibala assessment was performed once during their routine work schedule. For rotating-shift workers, the assessment was carried out twice, once during the day-shift cycle and again during the night-shift cycle, with data collection conducted at the end of each respective shift period to capture the influence of different work schedules on Jaṭharāgni status. Data were analyzed using the chi-squared test and the McNemar test. A *p*-value of < 0.05 was considered statistically significant. Cramer’s V test and Cohen’s test were used to calculate effect sizes for the chi-squared test and McNemar test, respectively.

**Results and discussion:**

The study indicated that most fixed-shift workers had an Agnibala status consistent with their Prakriti. In contrast, 61.7% of rotating-shift workers reported a change in their Agni status due to their job nature, with many shifting to Vishamagni during their night shifts. Statistical analysis confirmed that the nature of work significantly affects the Jaṭharāgni status, along with other factors such as Prakriti, habits, and diet. Based on these findings, it was recommended to adopt a diet and lifestyle plan tailored to Agni status to mitigate health impacts.

**Conclusion:**

This study findings suggest that rotating-shift work is associated with changes in the status of Agnibala, which may reflect disturbances in digestive and metabolic functioning. These observations indicate a possible relationship between shift work, circadian rhythm disruption, and digestive health. Although this study provides useful insights, further research involving larger populations, longitudinal designs, and molecular investigations is warranted to better understand these associations.

## 1 Introduction

Ayurveda put forward that the fundamental elements such as Doṣa, Dhātu, Mala, and Agni should be in proportional state for a person to maintain health (1). If any disequilibrium in these factors happens, the health will be impaired. Among these factors, Agni (digestive fire) is considered the root cause of all diseases (2). As per the classics, Agni is classified into four states as per the expression in physiological, digestive, and metabolic pattern based on the predominance of doshas (humors), such as Vishamāgni (irregular pattern of digestion commonly seen in Vata-predominant persons), Tīkṣṇāgni (excessively intense digestion commonly seen in Pitta-predominant persons), Mandāgni (slow digestion commonly seen in Kapha-predominant persons), and Samāgni (balanced state of digestion when the doshas are in equilibrium state) (3). Along with the predominance of tridoshas, Agni is also influenced by physiological factors such as Ritu (season), diurnal variation, Vaya (age), Prakṛti (basic constitution), morbid doṣas, quality and quantity of diet, and various aharaja (food-related factors), viharaja (activity-related factors), and manasika nidanas (psychological factors) (3). As the Agni is having a direct relation with physical and mental states of daily routine, a direct relation of status of Agni can be seen with nature of work of individuals (4).

Among the working population, 15–20% of the employees are engaged in shift work (5). The present-day lifestyle of a person is completely dependent on the time schedule of their work. Therefore, it may hamper the physical, physiological, and psychological health. Among the working population, fixed-shift workers are following a regular routine in lifestyle and diet, whereas rotating-shift workers are a group who are not able to follow a regular lifestyle. The prevalence of metabolic disorders is approximately 10% of the shift-working population (6). They are prone to the Agni-vitiating factors such as irregular dietary habits, suppression of urges, and sleep deprivation, making them prone to many metabolic disorders. The statement “All diseases begin in the gut,” quoted by the ancient Greek physician, Hippocrates, is being proved here. The vitiated Agni can cause imbalance in the gut microbiota (dysbiosis), which may lead to many health issues such as gastrointestinal disorders, autoimmune diseases, and metabolic disorders such as insulin resistance, diabetes, dyslipidemia, and metabolic syndrome, primarily due to disturbance in gut–brain endocrine axis (7). The growing importance of shift work in the modern society, as well as the alarming increase in the prevalence of metabolic diseases in them due to impairment of Agni, necessitates research in this area. The concept of Agni is personalized based on the factors such as Prakriti (constitution), Vaya (age), and Satmya (adaptability). By monitoring the Agnibala of a person, we can suggest personalized food and activities corresponding to the work habit following the strong principles of Agni in Ayurveda. Thus, we can give appropriate personalized dietary and activity plan schedule by monitoring the factors such as Prakriti along with Agni. There is no such study that has been done in this perspective.

Therefore, this study was undertaken to assess the status of Jaṭharāgni among rotating-shift workers and compare it with that of fixed-shift workers. It was hypothesized that rotating-shift workers would exhibit greater disturbances in Agnibala due to irregular work schedules and associated lifestyle factors. The findings of this study may contribute to a better understanding of the relationship between shift work and digestive health and help formulate appropriate dietary and lifestyle recommendations to reduce potential health impacts based on the status of Agni.

## 2 Materials and methods

The study was conducted in the Department of Samhita and Siddhanta at the Amrita School of Ayurveda in the academic year 2020–2021. Literature search was carried out in the classical texts of Ayurveda and Philosophy and relevant databases such as Google Scholar, PubMed, and Ayushportal.

### 2.1 Ethical committee approval and consent statement

The studies involving human participants were reviewed and approved by the Ethics Committee, Amrita Institute of Medical Sciences, Healthcare Education and Research, Kochi (IEC-AIMS-2019-AYUR-152) on 26 August 2019.

The study processes were explained to the participants and company CEOs, and consent forms prepared in English and Malayalam languages were provided before the data collection. Written informed consent was obtained from the participants to participate in this study.

### 2.2 Methodology

This study was carried out in four phases.

#### 2.2.1 Literature review

Literary searches were performed in different classical Ayurveda literatures and databases such as PubMed, Dhara, Scopus, Science Direct, and Google Scholar by using the keywords Agni in Ayurveda, Jaṭharāgni, Jaṭharāgni and digestion, digestive complaints of shift workers, and Jatharagni and shift workers. A total of 18,261 papers were found. After adding proper filters based on title relevance and abstract, 137 papers were found, and of those, the most relevant seven papers were selected. The primary study used a comparative cross-sectional design with a repeated assessment component among rotating-shift workers. The objective was to assess and compare the status of Agni among fixed-shift workers and rotating-shift workers employed in the Information Technology sector.

#### 2.2.2 Study population, sample size, and sampling procedure

The study samples were IT employees selected from UST Global and Applexus, Technopark, Thiruvananthapuram. The total population strength of IT employees of these selected companies was approximately 6,000. From this, a sample of 350 IT employees was selected by using Nassiuma’s formula, which is suitable for estimating sample size from a finite population when the coefficient of variation and margin of error are specified.

The formula used was: n = Nc2/[c2 + (N-1) e2], where:

- n = required sample size
- N = population size (6,000)
- C = coefficient of variation
- e = margin of error

A coefficient of variation of 30% (0.30) and a margin of error of 5% (0.05) were used, as recommended for social and health science research. Based on these values, the calculated sample size was approximately 350 participants.

A convenience sampling technique was used. Eligible participants were recruited voluntarily after obtaining informed consent through company communication channels and direct contact during the study period.

The participants were divided into two groups: Fixed-shift workers (*n* = 175): employees working in a regular fixed-shift schedule for at least 1 year.

Rotating-shift workers (*n* = 175): employees alternating between day shifts and night shifts on a monthly basis for at least 1 year.

Inclusion Criteria: The participants included in the study were apparently healthy male and female individuals who were willing to participate, aged between 25 and 45 years, and working in fixed or rotating shifts for at least 1 year.

Exclusion Criteria: Individuals with congenital abnormalities, diabetes mellitus, hypertension, or other chronic systemic diseases, those receiving long-term medication for systemic illnesses, those having recent history of acute illness, and pregnant and lactating women were excluded from the study. These exclusions were made to minimize factors that could independently influence Agni status.

#### 2.2.3 Assessment of Agni status

Agni status was assessed using a validated questionnaire developed by Singh et al. (27381899; PMCID: PMC5871217) (8). The instrument demonstrated acceptable internal consistency with a reported Cronbach’s alpha of 0.73. The questionnaire consists of 11 items, each providing four response options corresponding to the four classical states of Agni (3):

Samāgni: Balanced digestive and metabolic function.

Mandāgni: Diminished digestive capacity.

Tīkṣṇāgni: Excessively intense digestive activity.

Viṣamāgni: Irregular or fluctuating digestive activity.

Participants selected the response that best represented their habitual digestive experience.

After obtaining informed consent, questionnaires were administered in person by the investigator and provided standardized instructions for filling them. Participants completed the Agnibala assessment questionnaire in approximately 10–15 min. Completed forms were checked immediately for completeness. Forms with missing responses were returned to participants for clarification whenever possible. Questionnaires with substantial missing data were excluded from the analysis. For fixed-shift workers, Agni assessment was performed once during the study period. For rotating-shift workers, assessment was performed twice: During the day-shift month (5–8 days before completion of the shift cycle) and during the subsequent night-shift month (5–8 days before completion of the shift cycle). Data collection was carried out over 18 months. Approximately 20 rotating-shift workers and 20 fixed-shift workers were assessed during each cycle until the required sample size was achieved.

#### 2.2.4 Statistical analysis

Data were entered and analyzed using appropriate statistical software. The analysis focused on:

1. Association between participant characteristics and Agni status.
2. Association between nature of shift work and Agni status.
3. Changes in Agni status among rotating-shift workers during day and night shifts.
4. A chi-squared test was used to assess associations between categorical variables and status of Agni. The McNemar test was used to compare paired observations of Agni status among rotating-shift workers assessed during both day-shift and night-shift periods, as it is appropriate for analyzing changes in paired categorical data. Cramer’s V test and Cohen’s test were used to calculate the effect sizes for the chi-squared test and the McNemar test, respectively.

All statistical tests were two-tailed. A 95% confidence interval was considered, and a *p* < 0.05 was considered statistically significant.

## 3 Observations and results

Observations were made considering the variables age, gender, diet, habit, Prakṛti (constitution), and work shifts (Tables 1, 2) by using a 95% confidence interval for each variable. These variables were analyzed according to the status of Agnibala and the nature of work. Of the 175 fixed-shift workers, the status of Agni in the majority of individuals corresponded to their Prakṛti. Among the 175 rotating-shift workers, 108 (61.7%) showed a change in the status of Agni when they changed their day shifts to night shifts (Table 3).

**Table 1:** Variables and categorization.

Variables	Category	Number of fixed-shift workers	Number of rotating-Shift workers
Age	≤30 years	109	107
	30–35 years	32	42
	36–40 years	20	16
	41–45 years	14	10
Gender	Male	105	107
	Female	70	68
Diet	Vegetarian	45	47
	Mixed	130	128
Habit	Alcohol	14	19
	Smoking	12	4
	Both	32	35
	No habits	117	117
Prakruti	VK	40	54
	VP	28	36
	KP	24	27
	KV	32	21
	PV	28	15
	PK	23	22

**Table 2:** Categorization of fixed- and rotating-shift workers based on their type of Agni.

Agni	Regular		Rotating-shift workers			
			Day shift		Night shift	
	Number of individuals	%	Number of individuals	%	Number of individuals	%
Manda	12	6.9	20	11.4	32	18.3
Viṣāma	63	36	68	38.9	94	53.7
Sāma	94	53.7	78	44.6	32	18.3
Tīkṣṇa	6	3.4	9	5.1	17	9.7
Total	175	100	175	100	175	100

**Table 3:** Change of status of Agni in rotating-shift workers (day shift to night shift).

Change of Agni	Frequency	Percentage
No change	67	38.3
Mandagni to Viṣāmagni	9	5.1
Mandagni to Sāmagni	3	1.7
Mandagni to Tīkṣṇagni	1	0.6
Viṣāmagni to Mandagni	8	4.6
Viṣāmagni to Sāmagni	13	7.4
Viṣāmagni to Tīkṣṇagni	4	2.3
Sāmagni to Mandagni	17	9.7
Sāmagni to Viṣāmagni	38	21.7
Sāmagni to Tīkṣṇagni	9	5.1
Tīkṣṇagni to Viṣāmagni	4	2.3
Tīkṣṇagni to Sāmagni	2	1.1
Total	175	100

### 3.1 Age

Statistical analysis showed a chi-squared value of 2.122 and a *p*-value of 0.145, indicating no significant association between age and the status of Agnibala.

### 3.2 Gender

The chi-squared test yielded a χ^2^ value of 0.563 and a *p*-value of 0.453, indicating no significant association between gender and the status of Agnibala.

### 3.3 Diet

Statistical analysis showed a chi-squared value of 3.625 and a *p*-value of 0.05, indicating a significant association between dietary pattern and the status of Agni when the participants changed their shifts from day shifts to night shifts. The effect size calculated by Cramer’s V test (*V* = 0.144) indicates that even though a statistically significant association is observed between Agni and diet, the strength of association is weak (Table 4).

**Table 4:** Variation of Agni in rotating-shift workers related to diet.

Change in Agni	Vegetarian	Diet		%	Total
		%	Mixed		
No change	13	19.4	54	80.6	67
Manda to Viṣāma	2	22.2	7	77.8	9
Manda to Sāma	0	0.0	3	100.0	3
Manda to Tīkṣṇa	0	0.0	1	100.0	1
Viṣāma to Manda	2	25	6	75.0	8
Viṣāma to Sāma	7	53.8	6	46.2	13
Viṣāma to Tīkṣṇa	0	0.0	4	100.0	4
Sāma to Manda	5	29.4	12	70.6	17
Sāma to Viṣāma	12	31.6	26	68.4	38
Sāma to Tīkṣṇa	5	55.6	4	44.4	9
Tīkṣṇa to Viṣāma	1	25	3	75	4
Tīkṣṇa to Sāma	0	0.0	2	100.0	2
Total	47	26.9	128	73.1	175

Chi-squared value - 3.625 *P*-value = 0.05 *V* = 0.144.

### 3.4 Habits

Statistical analysis showed no significant association between habits, the status of Agni, and the nature of work. The *p*-values obtained from the chi-squared test were 0.410, 0.134, and 0.567, respectively, for the categories of habits, namely, alcohol, smoking, and both habits.

### 3.5 Prakṛti

This includes six groups: Those who are having VataKapha prakrti, KaphaVata prakrti, VataPitta prakrti, PittaVata prakrti, PittaKapha prakrti, and KaphaPitta prakrti. The chi-squared test showed a χ^2^ value of 5.225 and a *p*-value of 0.02, indicating a significant association between Prakṛti, the status of Agni, and the nature of work. The effect size calculated by Cramer’s V test (*V* = 0.77) indicates that even though statistically significant association is observed between the status of Agni and Prakriti, the strength of association is not so strong (Table 5).

**Table 5:** Change in the status of Agni in rotating-shift workers based on prakrti.

Change in the status of Agni	Prakṛti												Total
	Vata-Kapha	%	KaphaVata	%	VataPitta	%	PittaVata	%	PittaKapha	%	Kapha-Pitta	%	
No change	22	32.8	13	19.4	15	22.4	9	13.4	4	6.0	4	6.0	67
Manda to Vishama	6	66.7	1	11.1	0	0.0	0	0.0	0	0.0	2	22.2	9
Manda to Sama	0	0.0	1	33.3	0	0.0	0	0.0	0	0.0	2	66.7	3
Manda to Tīkṣṇa	0	0.0	0	0.0	0	0.0	0	0.0	1	100.0	0	0.0	1
Vishama to Manda	4	50.0	2	25.0	1	12.5	0	0.0	1	12.5	0	0.0	8
Vishama to Sama	4	30.8	2	15.0	2	15.4	2	15.4	2	15.4	1	7.7	13
Vishama to Tīkṣṇa	0	0.0	1	25.0	3	75.0	0	0.0	0	0.0	0	0.0	4
Sama to Manda	3	17.6	8	47.1	0	0.0	1	5.9	0	0.0	5	29.4	17
Sama to Vishama	15	39.5	7	18.4	5	13.2	3	7.9	4	10.5	4	10.5	38
Sama to Tīkṣṇa	0	0.0	1	11.1	0	0.0	3	33.3	3	22.3	2	22.2	9
Tīkṣṇa to Vishama	0	0.0	0	0.0	0	0.0	3	75.0	0	0.0	1	25.0	4
Tīkṣṇa to Sama	0	0.0	0	0.0	1	50.0	0	0.0	0	0.0	1	50.0	2
Total	54	30.9	36	20.6	27	15.4	21	12.0	15	8.6	22	12.6	175

Chi-squared value = 5.225 *p* = 0.02 *V* = 0.077.

### 3.6 Nature of work

Fixed-shift workers generally exhibited Agni corresponding to their Prakṛti. Rotating-shift workers showed changes in the status of Agni when on day and night shifts (Table 6).

**Table 6:** Change in the status of Agni in rotating-shift workers based on the nature of work.

Change of Agni		Night shift				Total
		Manda	Vishama	Sama	Tīkṣṇa	
Day shift	Manda	7	9	3	1	20
	Vishama	8	43	13	4	68
	Sama	17	38	14	9	78
	Tīkṣṇa	0	4	2	3	9
Total		32	94	32	17	175

McNemar test *P* = 0.001 *W* = 0.396.

#### 3.6.1 Mandāgni in day shifts

Of the 175 rotating-shift workers, 20 workers had Mandāgni during the day shift. Among them, 7 (35%) showed no change in their Agni status, 9 (45%) shifted to Viṣamāgni, 3 (15%) shifted to Samāgni, and 1 (5%) shifted to Tīkṣṇāgni during the night shift.

#### 3.6.2 Viṣamāgni in day shifts

Of the 175 rotating-shift workers, 68 workers had Viṣamāgni during the day shift. Among them, 43 (63.2%) showed no change, while 8 (11.7%) shifted to Mandāgni, 13 (19.1%) shifted to Samāgni, and 4 (5.8%) shifted to Tīkṣṇāgni during the night shift.

#### 3.6.3 Samāgni in day shifts

Of the 175 rotating-shift workers, 78 workers had Samāgni during the day shift. Among them, 14 workers showed no change, while 17 workers shifted to Mandāgni, 38 workers shifted to Viṣamāgni, and 9 workers shifted to Tīkṣṇāgni during the night shift.

#### 3.6.4 Tīkṣṇāgni in day shifts

Of the 175 rotating-shift workers, 9 workers had Tīkṣṇāgni during the day shift. Among them, 3 workers showed no change, 4 workers shifted to Viṣamāgni, and 2 workers shifted to Samāgni during the night shift. No worker shifted to Mandāgni.

The McNemar test (*p* < 0.001) indicated a statistically significant association between shift work and changes in the status of Agni. The effect size for the association between shift work and Agni was calculated using Cohen’s test, and the value was w = 0.396, which indicates a greater effect.

Overall, the analysis showed variations in Agni status according to diet, Prakṛti, and the nature of work, whereas age, gender, and habits did not show a significant association (Table 7**)** The effect size was also calculated for the variables that showed an association, and it was found out that the association between shift work and Agni is relatively stronger than the association between Agni with diet and Prakṛti. Therefore, it is clear that the change in shift has an impact on the status of Agni.

**Table 7:** Table showing association of all variables with Agnibala.

Variable	χ^2^ value	*p*-value	Effect size (Cramer’s V)	Interpretation
Age	2.122	0.145		Not significant
Gender	0.563	0.453		Not significant
Diet	3.625	0.05	*V* = 0.144	Borderline significant with weak association
Habit	0.410, 0.134, and 0.567	0.915		Not significant
Prakruti	5.225	0.02	*V* = 0.077	Significant with weak association
Variable		P Value	Effect size (Cohen’s test)	Interpretation
Shift work McNemar	27.56 (Chi-squared value)	0.001	*W* = 0.396	Significant with relatively strong association

Used 95% confidence intervals for all variables.

## 4 Discussion

This study evaluated the association of Agnibala with variables, namely age, gender, diet, habits, Prakṛti, and the nature of work. These findings showed significant variations in Agni status with respect to diet, Prakṛti, and the nature of work, whereas age, gender, and habits did not show a statistically significant association. These observations suggest that factors directly influencing digestive physiology and biological rhythms may have a greater impact on Agni than other general demographic factors. Although the association was statistically significant for diet and Prakriti, the statistical analysis indicated a weak association for both. This suggests that while dietary pattern and Prakriti contribute to variations in Agni status, it may not be the sole determining factor. However, the association reported for ‘nature of work’ showed a relatively stronger relationship with Agni status, suggesting that shift work had a significant impact on digestive function.

By analyzing the data, it was observed that among the workers having a change in the status of Agni, the highest number of employees shifted to Viṣamāgni. The second largest group of employees shifted to Mandāgni during their night shifts. According to Ayurveda, impairments due to shift work, such as deranged food habits, sleep habits, and other lifestyle disturbances, may vitiate Doṣas, especially Vāta (3). Therefore, the predominance of change from Samāgni to Viṣamāgni among workers during night shifts may be interpreted on this basis. Similarly, improper sleep patterns associated with shift work often lead workers to sleep during the daytime. Such sedentary patterns of Āhāra and Vihāra are considered factors that may promote Kapha vṛddhi (3), which may theoretically contribute to the development of Mandāgni (3). Among the rotating-shift workers, 67 individuals did not show any change in Agni status. This may indicate that some workers were able to maintain their digestive balance despite shift-related disturbances. They might have followed proper physical exercise (3) and attempted to maintain regular timings for food and daily activities. However, these factors were not directly assessed in this study. Many recent studies are going on to address the efficacy of physical activity or dietary interventions during shift work (9). This finding also suggests that shift work may not affect all individuals equally and that individual adaptability or other lifestyle factors may play a role in maintaining Agni balance (10).

As far as shift work is concerned, previous studies have reported negative effects on worker health, possibly due to its impact on sleep–wake cycles, eating habits, exercise habits, thermogenesis, hormone secretion, and blood pressure level. They have also reported a higher prevalence of gastrointestinal complaints and metabolic disturbances among shift workers compared to day workers (11). The findings of this study are consistent with these observations, as changes in Agni status were more commonly observed among rotating-shift workers during night shifts. Many previous research studies have suggested that shift work can disrupt the circadian rhythm. Circadian disruption has been associated with gastrointestinal disturbances and metabolic disorders (12).

As per the classics, Prakriti (constitution) is a factor that influences the factors such as Agni (digestion), Koshta (bowel), and Vyadhikshamatva (immunity). Many recent studies have highlighted Ayurvedic Prakṛti phenotyping and its relationship with gut microbiota. The predominant microbial community has been reported to vary according to the predominant Doṣa Prakṛti of an individual. Although Prakṛti remains relatively stable throughout life, gut microbiota composition may vary under the influence of several factors that may also influence Jaṭharāgni (3). However, this study did not assess gut microbiota, endocrine parameters, or circadian biomarkers. Therefore, these mechanisms are only possible explanations derived from previous literature and cannot be confirmed by the present findings.

As the concept of Agni is personalized based on Prakriti and other factors, this study suggests a P4 approach to occupational medicine. By analyzing the change of Agni, possible metabolic impairments can be predicted, and preventive measures can be adopted at the earliest. Therefore, personalized care can be given to prevent the disorders and for the optimization of individual health (13).

## 5 Suggestions for maintaining Agni

Based on classical Ayurvedic principles, the following dietary and lifestyle recommendations (1) may be considered:

For Samāgni

- Maintain regular meal timings.
- Follow a balanced diet and daily routine.
- Continue healthy lifestyle practices to preserve normal digestive function.

For Viṣamāgni

- Prefer warm, freshly prepared, unctuous, sour, and salty foods.
- Avoid cold, refrigerated, and difficult-to-digest foods.
- Maintain regular timings for food, sleep, and waking.
- Practice mental relaxation and avoid excessive stress and anxiety.
- Protect the body from excessive cold exposure and include good-quality oils in the diet.

For Mandāgni

- Avoid heavy, excessively unctuous, sweet, sour, and salty foods.
- Include pungent, bitter, and astringent foods.
- Engage in regular physical exercise.
- Use honey appropriately as advised in Ayurvedic texts.
- Avoid cold foods and excessive exposure to cold environments.

For Tīkṣṇāgni

- Prefer cooling, nourishing, and unctuous foods.
- Include milk and ghee in the diet when appropriate.
- Practice cooling and calming activities such as yoga and prāṇāyāma.
- Avoid excessively spicy, hot, and irritant foods.
- Follow a Pitta-pacifying lifestyle and daily routine.

## 6 Conclusion

This study assessed the status of Agni among fixed-shift and rotating-shift IT employees and explored its association with selected factors such as Prakṛti and dietary habits. These findings indicate that Viṣamāgni was the most common change observed among rotating-shift workers during night-shift periods, while a smaller proportion exhibited a transition to Mandāgni. In contrast, a considerable number of workers who reported healthier dietary habits and regular lifestyle practices tended to maintain a stable Agni status irrespective of their work schedule. The results highlight the practical importance of maintaining appropriate dietary habits, regular meal timings, physical activity, and healthy sleep practices among shift workers. Further large-scale, multicenter, and longitudinal studies incorporating objective physiological and metabolic assessments are required to better understand the relationship between shift work, Agni, and metabolic health. This study is preliminary evidence supporting Ayurvedic concepts of Agni in personalized and preventive practices and thereby enhances the health status of an individual.

### 6.1 Limitations of the study

The findings of this study should be interpreted in light of several limitations. First, the assessment of Agni was based on a self-reported questionnaire, and therefore the responses depended on the participants’ subjective perception of their digestive status. This may have introduced reporting bias. Since some questions required participants to report their usual digestive experiences and lifestyle practices, the possibility of recall bias cannot be excluded.

Second, due to entry restrictions in several IT companies, a portion of the data had to be collected through Google Forms and telephonic conversations. Although efforts were made to ensure uniform data collection, variations in the mode of questionnaire administration may have influenced participant responses.

Third, obtaining permission from human resource managers of IT companies was challenging because of organizational policies and employee workload. As a result, participant recruitment was limited to selected companies.

Fourth, the study was conducted among employees of selected IT companies in Technopark, Thiruvananthapuram, representing a single geographic setting. Therefore, the findings may not be fully representative of shift workers in other industries, regions, or populations.

Fifth, although rotating-shift workers were assessed during both day-shift and night-shift periods, the study was essentially observational and cross-sectional in nature. Consequently, causal relationships between shift work and changes in Agni status cannot be established.

Finally, the sample size was constrained by the available study period and accessibility of participants. Therefore, the generalizability of the findings may be limited.

Future studies involving larger, multicentric populations and objective physiological assessments are required to validate and extend the present observations.

### 6.2 Further scope of the study

- After validating the results using large sample sizes, the molecular changes in the digestive system corresponding to shift work can be further studied.
- Detailed study of each variable can be conducted.
- Studies on gut transit time can be conducted to analyze the status of Agni.
- A study on Agni can be used as a tool to diagnose a premetabolic disorder stage and can adopt preventive measures from metabolic diseases.

## Data Availability

The raw data supporting the conclusions of this article will be made available by the authors, without undue reservation.
